# Cross-variant protection against SARS-CoV-2 infection in hamsters immunized with monovalent and bivalent inactivated vaccines

**DOI:** 10.7150/ijbs.72109

**Published:** 2022-07-13

**Authors:** Zi-Wei Ye, Yilan Fan, Kaiming Tang, Chon Phin Ong, Cuiting Luo, Hon-Lam Chung, Tsun-Lam Leong, Ronghui Liang, Wai-Yin Lui, Runhong Zhou, Yun Cheng, Lu Lu, Pak-Hin Hinson Cheung, Jasper Fuk-Woo Chan, Zhiwei Chen, Kwok-Yung Yuen, Shuofeng Yuan, Kelvin Kai-Wang To, Dong-Yan Jin

**Affiliations:** 1 Department of Microbiology, Li Ka Shing Faculty of Medicine, The University of Hong Kong, Pokfulam, Hong Kong; 2 School of Biomedical Sciences, Li Ka Shing Faculty of Medicine, The University of Hong Kong, Pokfulam, Hong Kong; 3 State Key Laboratory of Emerging Infectious Diseases, Li Ka Shing Faculty of Medicine, The University of Hong Kong, Pokfulam, Hong Kong

**Keywords:** COVID-19, Inactivated vaccine, Neutralizing antibodies, Immune protection, SARS-CoV-2 variants of concern

## Abstract

Rapid development and successful use of vaccines against SARS-CoV-2 might hold the key to curb the ongoing pandemic of COVID-19. Emergence of vaccine-evasive SARS-CoV-2 variants of concern (VOCs) has posed a new challenge to vaccine design and development. One urgent need is to determine what types of variant-specific and bivalent vaccines should be developed. Here, we compared homotypic and heterotypic protection against SARS-CoV-2 infection of hamsters with monovalent and bivalent whole-virion inactivated vaccines derived from representative VOCs. In addition to the ancestral SARS-CoV-2 Wuhan strain, Delta (B.1.617.2; δ) and Theta (P.3; θ) variants were used in vaccine preparation. Additional VOCs including Omicron (B.1.1.529) and Alpha (B.1.1.7) variants were employed in the challenge experiment. Consistent with previous findings, Omicron variant exhibited the highest degree of immune evasion, rendering all different forms of inactivated vaccines substantially less efficacious. Notably, monovalent and bivalent Delta variant-specific inactivated vaccines provided optimal protection against challenge with Delta variant. Yet, some cross-variant protection against Omicron and Alpha variants was seen with all monovalent and bivalent inactivated vaccines tested. Taken together, our findings support the notion that an optimal next-generation inactivated vaccine against SARS-CoV-2 should contain the predominant VOC in circulation. Further investigations are underway to test whether a bivalent vaccine for Delta and Omicron variants can serve this purpose.

## Introduction

The ongoing pandemic of COVID-19 caused by infection with SARS-CoV-2 has caused substantial morbidity and claimed over 4 million lives in the past two years. Strict public health measures and vaccination campaign have helped to curtail the pandemic 1, 2. Despite these efforts, SARS-CoV-2 has evolved to acquire several high-effect mutations, which gave rise to the variants of concern (VOCs), including the Alpha (B.1.1.7), Beta (B.1.351), Gamma (P.1), Delta (B.1.617.2) and Omicron (B.1.1.529) 3. Compared to the ancestral Wuhan strain, all these VOCs already contain another high-effect mutation D614G in the spike region, which increases SARS-CoV-2 fitness and transmissibility 4. The Alpha variant was first identified in the United Kingdom and found to have increased transmissibility 5, while the Beta variant, first discovered in South Africa, was weakly neutralized by plasma from convalescent patients or vaccinees 6. The Gamma variant initially found from Brazil was circulating in South America, showing a high degree of immune evasion 7. Unlike the Alpha variant, the immune-evasive Beta and Gamma variants had no chance to become the predominant variant at the global scale 8. In contrast, the highly transmissible Delta variant arisen from India quickly outcompeted the Alpha variant to dominate globally 9. The continuous emergence of new SARS-CoV-2 VOCs has sustained its spread in human populations 10. The most recent SARS-CoV-2 VOC designated Omicron (B.1.1.529) contains more than 30 changes in its spike protein including 15 substitutions at the receptor-binding domain (RBD) (Figure S1). This variant was initially reported in South Africa and Hong Kong 11, 12. Emerging evidence suggests that the Omicron variant is highly transmissible, highly immune-evasive but less pathogenic 12, resulting in a high rate of generally mild reinfection and breakthrough infection 11-18.

Before SARS-CoV-2 evolves to a sufficiently attenuated post-pandemic version, vaccination remains to be the most effective measure to reduce virus transmission and to prevent severe disease 19. Currently, four types of SARS-CoV-2 vaccines, namely inactivated, adenovirus-vectored, mRNA and protein subunit vaccines, have been used in humans 2. Among them, both adenovirus-vectored and mRNA vaccines are first-in-class to receive approval for emergency use. Although their protective efficacies might not be as high as those of the other three types of vaccines, the whole-virion inactivated vaccines have been massively produced and distributed, particularly to resource-limiting countries 20. Adenovirus-vectored vaccines have also been widely and successfully used 21, but are overshadowed by rare thrombotic complications ascribed to vaccine-induced antibodies against platelet factor 4 22. Protein subunit vaccines are highly efficacious, but their mass production might be technically more challenging 23. mRNA vaccines, which represent a new class of next-generation vaccines, have been shown to be highly efficacious and are capable of inducing neutralizing antibodies and cell-mediated immunity to exceedingly high levels 24, 25. They are therefore considered the frontrunners that hold great promises in bringing an end to the pandemic through vaccination. Notably, mRNA vaccines are known to provide good or acceptable protection against some VOCs 26, 27. However, waning of neutralizing antibodies a few months after vaccination and vaccine evasion by newly emerged VOCs are the two major challenges that prevent the SARS-CoV-2 vaccination program from achieving further success 2, 13, 28. Up to date, all vaccines approved for human use are based on the ancestral Wuhan strain in 2019 or its closest descendent instead of the circulating strains. In view of this, the development of variant-targeting vaccines for primary and booster injection might be necessary. Particularly, it is not known whether a bivalent vaccine might be a viable option. Neither is it clear whether vaccines directed against a prevalent SARS-CoV-2 variant could cross-protect other variants that circulate concurrently or later. These questions might be best addressed with inactivated vaccines since variant-specific versions can be made immediately after the isolation of variant strains.

In this study, we investigated cross-variant protective efficacies of variant-specific inactivated vaccines against SARS-CoV-2. Vaccines specifically targeting Delta variant and another less common variant named Theta or P.3 with an N501Y mutation in the spike region (Figure S1) were tested for the ability to provide homotypic and heterotypic protection against SARS-CoV-2 infection in hamsters. More importantly, we assessed the efficacies of bivalent inactivated vaccines, which contained ancestral strain plus either Delta or Theta variant, against emerging SARS-CoV-2 variants. These were then administered to the hamsters to study the efficacy and immunogenicity of the bivalent inactivated vaccine. The vaccinated hamsters were further challenged with the prevailing variants, including the Omicron or Delta variant, to determine whether the bivalent vaccines can provide better protection.

## Results

### Humoral immune responses triggered by different inactivated vaccines

**Figure 1:**
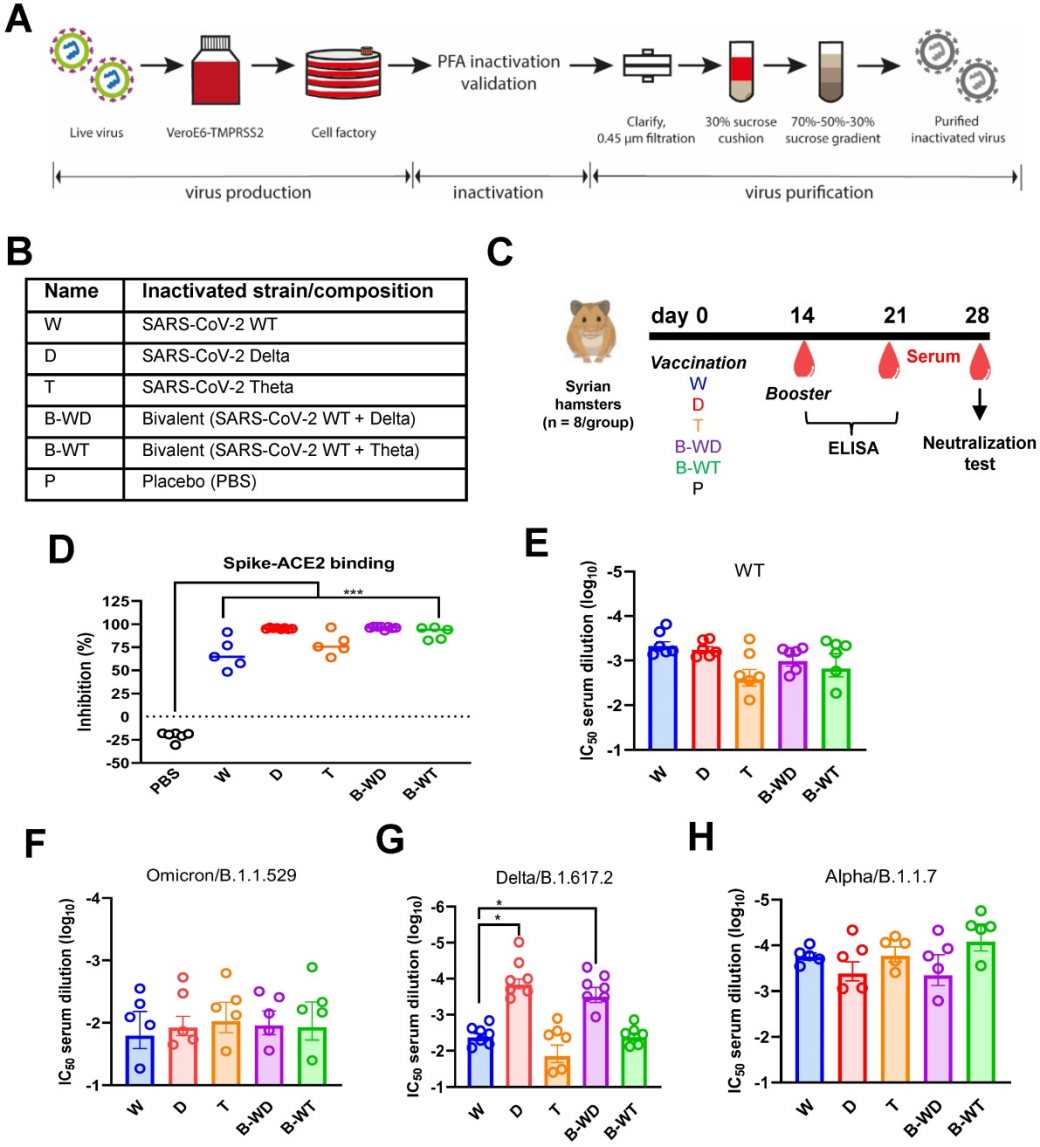
Humoral immune responses in hamsters vaccinated with inactivated viruses. (**A**) Schematic diagram showing the procedure for generation of the inactivated vaccine in this study. (**B**) Abbreviations used for the indicated inactivated vaccine groups. (**C**) Vaccination scheme. Hamsters were intramuscularly injected with various inactivated SARS-CoV-2 strains or placebo control (PBS) at days 0 and 14. Blood samples were collected at days 0, 14, 21 and 28 for ELISA or neutralization tests. (**D**-**H**) Neutralization activity of serum samples collected at day 28 post-vaccination from hamsters immunized with different inactivated vaccines. The neutralization activity of the serum samples collected at day 28 post-vaccination were analyzed by sVNT or microneutralization assay with the indicated VOCs of SARS-CoV-2. (**D**) cPass SARS-CoV-2 neutralization antibody detection. The broken line indicates detection limit. (**E-H**) Microneutralization assay with sera from the indicated groups collected at day 28 post-vaccination against WT (**E**) and the following three VOCs of SARS-CoV-2: Omicron/B.1.1.529 (**F**), Delta/B.1.617.2 (**G**) and Alpha/B.1.1.7 (**H**).

Different forms of monovalent and bivalent inactivated SARS-CoV-2 vaccines were generated in Vero-E6-TMPRSS2 cells and purified through ultracentrifugation (Figure 1A and B). Three SARS-CoV-2 strains (Figure S1) were chosen for comparison: the ancestral Wuhan strain herein designated wild type (WT), the Delta variant, which was highly prevalent worldwide at the beginning of our study 9 and the Theta variant, which was initially found in the Philippines and is relatively rare 29. The expression of spike protein from Vero-E6-TMPRSS2 cells infected with different viruses was verified by Western blot analysis (see Figure S2 for one example). Hamsters were intramuscularly injected with PBS (placebo control, P) or inactivated version of WT (W), Delta (D) or Theta (T) strains of SARS-CoV-2. The two variants were paired up with the Wuhan strain to make a bivalent version of the inactivated vaccine (Figure 1B), abbreviated B-WD or B-WT. Vaccines (3 µg/dose) were mixed with alum adjuvant for injection. At 14 days post-vaccination, a booster shot (3 µg/dose) was administered to each group. Wild-type receptor-binding domain (RBD)-specific antibody responses were evaluated by ELISA at days 14, 21 and 28 (Figure S3). After the primary immunization, all vaccinated hamsters had developed humoral immune responses, which became more pronounced after the booster shot (Figure S3). Collectively, these results suggested that all five vaccines were capable of inducing RBD -specific humoral immune responses in hamsters.

Neutralizing antibodies (NAbs) are the best correlate of immune protection against SARS-CoV-2 30-32. In view of this, SARS-CoV-2 surrogate virus neutralization test (sVNT) 33 was performed to assess the neutralizing capabilities of sera collected 4 weeks after vaccination (Figure 1D). The results showed that sera from all vaccinated groups were competent in inhibiting RBD-ACE2 interaction. To further compare the neutralizing performance of the candidate vaccines, microneutralization assay against different SARS-CoV-2 variants was conducted (Figure 1E-H). All vaccines tested induced relatively high levels of NAbs against the ancestral Wuhan strain and the Alpha variant (Figure 1E and H). Reduced neutralizing titers against the Omicron variant were observed in all groups (Figure 1F), which is in general agreement with previous findings 13, 15. When neutralization of the Delta variant was assayed, the cognate D and B-WD groups demonstrated noticeable neutralizing potencies over the other three groups (Figure 1G). Combined, these results suggested that the three monovalent and the two combinatorial bivalent inactivated vaccines were all capable of conferring cross-variant protection against SARS-CoV-2. Their cross-protective potencies diminished at the Delta variant and more remarkably at the Omicron variant.

### Better protection against the Omicron variant by the bivalent B-WT vaccine

**Figure 2:**
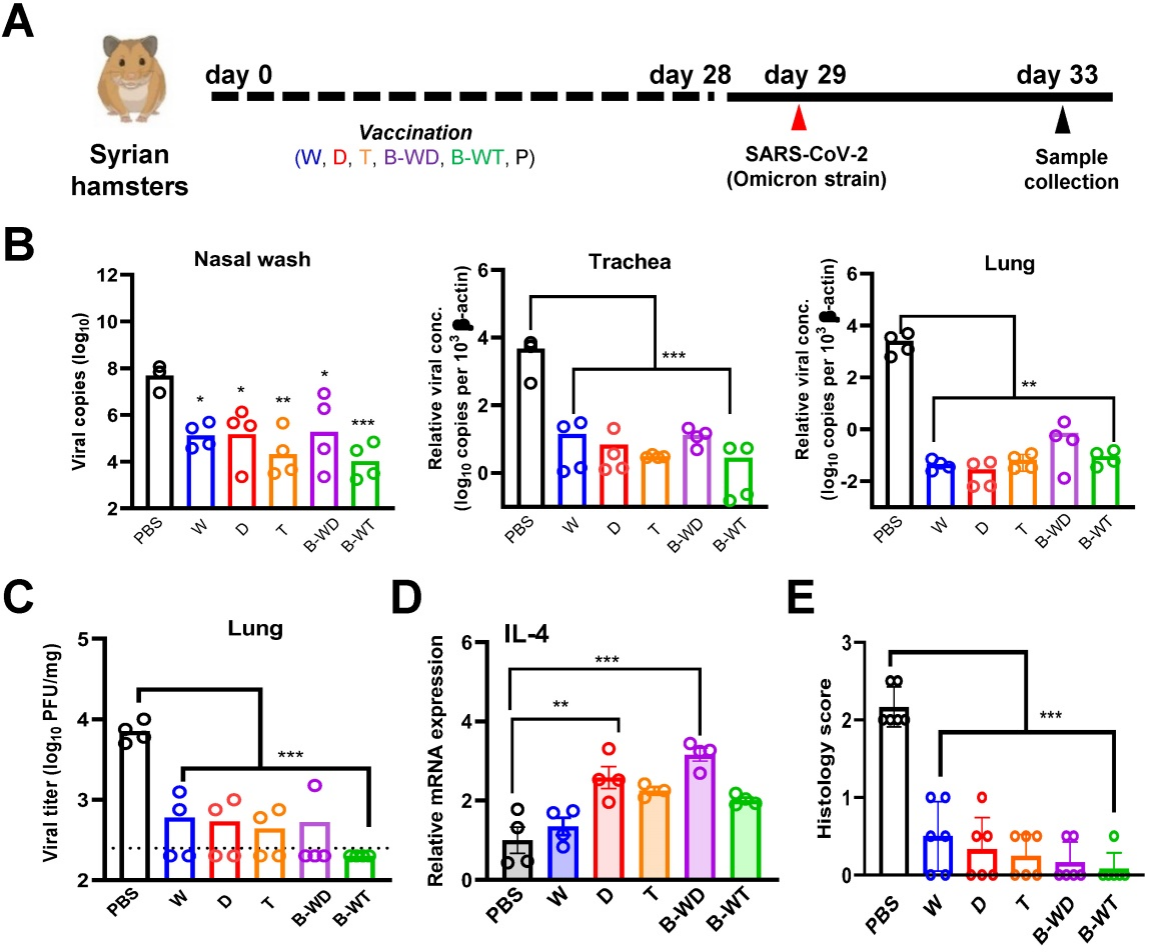
Vaccination with inactivated SARS-CoV-2 VOCs confers protection to infection of hamster lungs with Omicron variant. (**A**) Virus challenge scheme. Vaccinated hamsters were intranasally inoculated with 10^5^ PFU (in 50μl) of SARS-CoV-2 Omicron variant. At 4 dpi (i.e. 33 days after vaccination), viral titers in hamster nasal wash, tracheal and lung samples were quantitated by RT-qPCR (**B**). (**C**) Infectious viral titers were quantified with plaque assay in Vero-E6-TMPRSS2 cells. The broken line indicates the limit of detection. (**D**) IL-4 gene expression in hamsters infected with SARS-CoV-2 Omicron variant. Transcripts of representative cytokines in the lung tissue homogenates of the indicated groups were quantitated by RT-qPCR. Results are shown as means ± SEM. Statistical analysis was performed by Student's t test (*: P < 0.05; **: P < 0.01; ***: P < 0.001). (**E**) Pathological changes in Omicron-infected lungs from the indicated vaccinated groups (***: P < 0.001).

**Figure 3:**
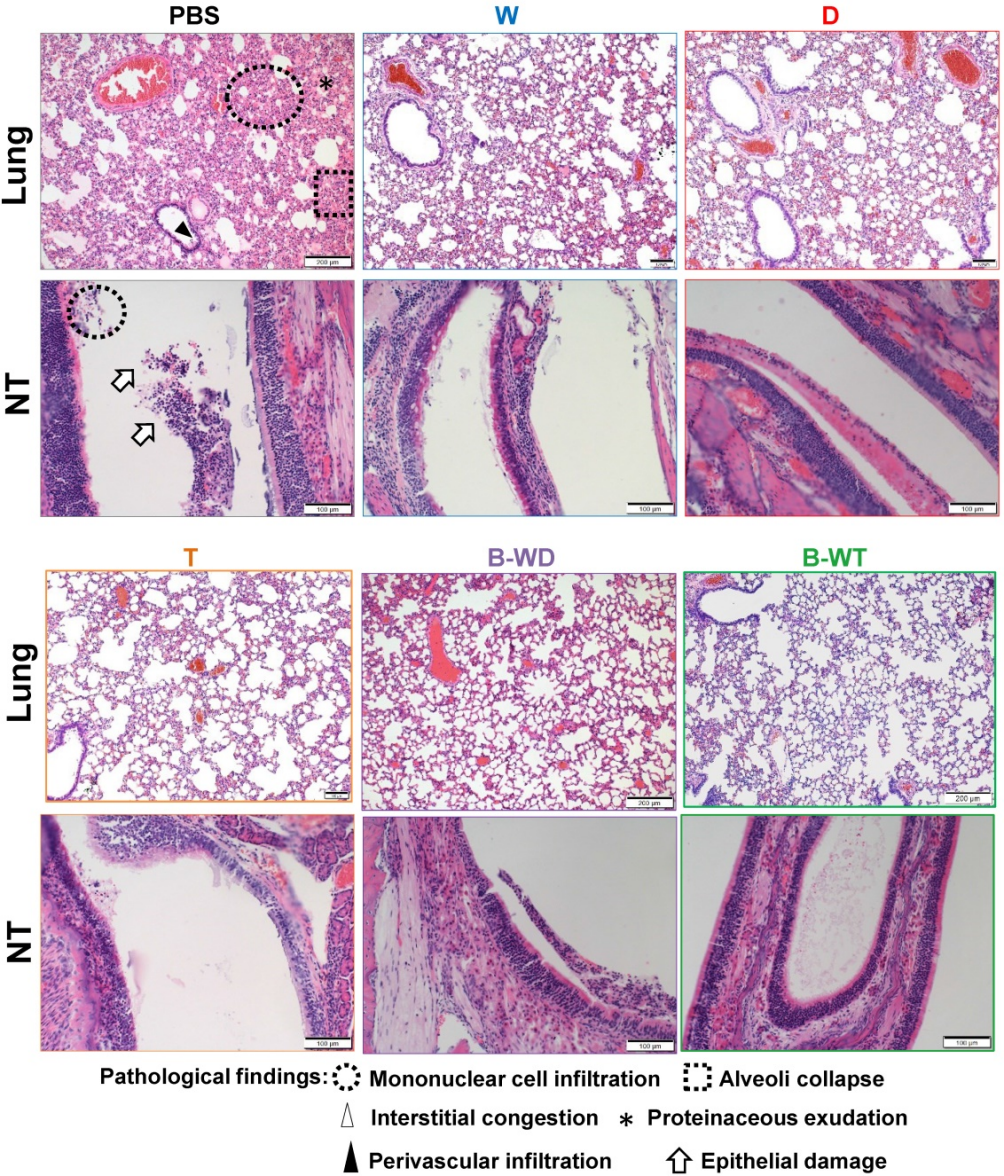
Histopathological changes in nasal turbinates and lungs of Omicron variant-infected hamsters vaccinated with the indicated inactivated vaccines. Representative images of nasal turbinate (NT) and lung sections of Omicron-infected hamsters were shown. Histopathological changes in nasal turbinate and lung tissues were examined by hematoxylin and eosin staining.

To further compare the *in vivo* protective efficacies of different inactivated vaccines, we infected the vaccinated hamsters with the Omicron variant (Figure 2A). A significantly decreased viral load was seen in nasal wash, tracheal and lung samples collected from all five vaccinated groups (Figure 2B and C), among which the most pronounced decrease was observed with the B-WT bivalent vaccine group (Figure 2B). The observation was in line with plaque assay, with the least live virus being detected in the lung sample from the B-WT-vaccinated group (Figure 2C). We also evaluated cytokine gene expression levels in vaccinated hamsters after viral challenge. Proinflammatory cytokine interleukin (IL)-4 was selected for analysis since it might facilitate mucosal and T cell immunity 34. The mRNA expression levels of IL-4 markedly increased in the D and B-WD groups (Figure 2D). In addition, histopathological evaluation in lungs and nasal turbinates also showed amelioration of tissue damage in vaccinated hamsters. Whereas the least damage was observed in the B-WT-vaccinated hamsters, residual pathological changes and trace of inflammation were marginally noticeable in the hamsters receiving the W vaccine (Figure 2E and Figure 3). Taken together, our results indicated that all vaccines ameliorated tissue injury upon Omicron variant infection, among which the most efficient protection seemed to be offered by the B-WT vaccine, given that an Omicron-specific vaccine was not yet available.

### Bivalent B-WD vaccine offers optimal protection against the Delta variant

**Figure 4:**
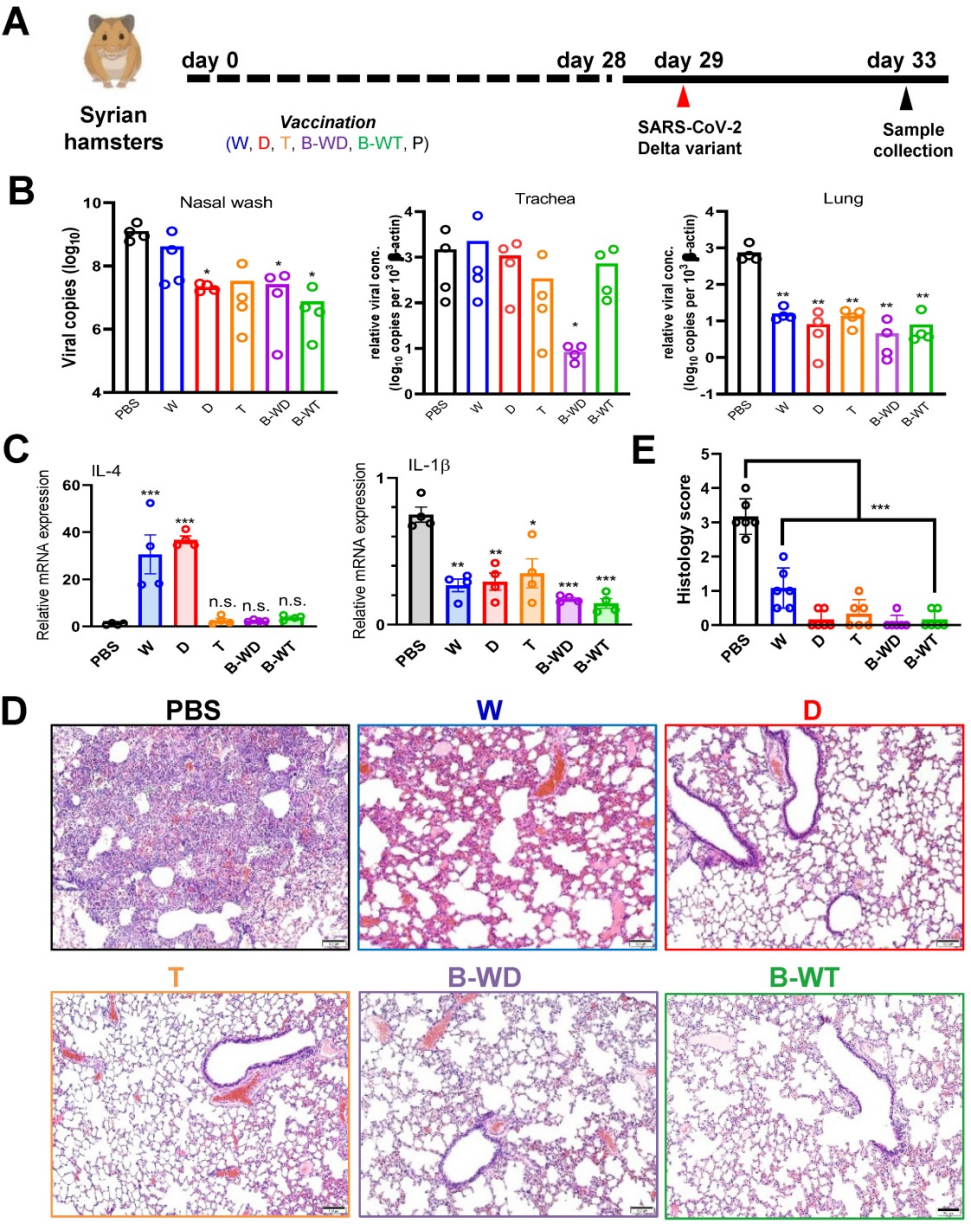
Vaccination with the indicated inactivated viruses confers protection to infection of hamster lung with SARS-CoV-2 Delta strain. (**A**) Virus challenge scheme. Vaccinated hamsters were intranasally inoculated with 10^5^ PFU (in 50μl) of SARS-CoV-2 Delta variant. At 4 dpi (i.e., 33 days after vaccination), viral titers in hamster nasal wash, tracheal and lung samples were quantitated by RT-qPCR (**B**). (**C**) Cytokine gene expression in Delta variant-infected hamsters. Transcripts of representative chemokines and cytokines in the lung tissue homogenates of the indicated groups were quantitated by RT-qPCR. Results are shown as means ± SEM. Statistical analysis was performed by Student's t test and comparison was made to the PBS group (n.s.: not significant; *: P < 0.05; **: P < 0.01; ***: P < 0.001). (**D**) Impact of vaccination on histopathological changes in lungs of SARS-CoV-2 Delta variant-infected hamsters. Lung histopathological changes of each vaccinated hamster group at 4 dpi. Representative lung tissue sections were stained with hematoxylin and eosin. Bar, 100 μm. (**E**) Pathological changes in Delta-infected lungs from indicated vaccinated groups, scored as described in Methods (**: P < 0.01).

The Delta variant was the predominant SARS-CoV-2 strain circulating worldwide when we started our study 9. Since existing mRNA vaccines exhibit fair protection against the Delta variant 17, 35, 36, particularly after three injections 27, 37, 38, it remains controversial whether a Delta-specific vaccine might be necessary. To shed light on this, we performed infection study with the vaccinated hamsters and the Delta variant (Figure 4A). Marginal changes in viral load were seen in trachea except for the B-WD group. In contrast, a significant decrease in the viral load was observed in the nasal wash and lung tissue of all vaccinated hamsters (Figure 4B). In particular, viral loads in the tracheal and lung tissues of B-WD-vaccinated hamsters dropped to an undetectable level, suggestive of no viral replication. In addition to IL-4 that might have adjuvant activity 34, IL-1β that is a key mediator of proinflammatory response 39 was also selected for analysis after viral challenge. Whereas the levels of IL-4 mRNA were markedly elevated in the W- and D-vaccinated groups, a remarkable drop in IL-1β transcripts was seen in most groups (Figure 4C). Additionally, noticeable amelioration in lung damage was observed in all groups except for the W group (Figure 4D, E). Collectively, these results suggested that tissue damage caused by infection with the Delta strain was ameliorated in all vaccinated groups, with optimal protection offered by immunization with the cognate D and B-WD vaccines.

### Good protective efficacies of monovalent and bivalent inactivated vaccines against the Alpha variant

**Figure 5:**
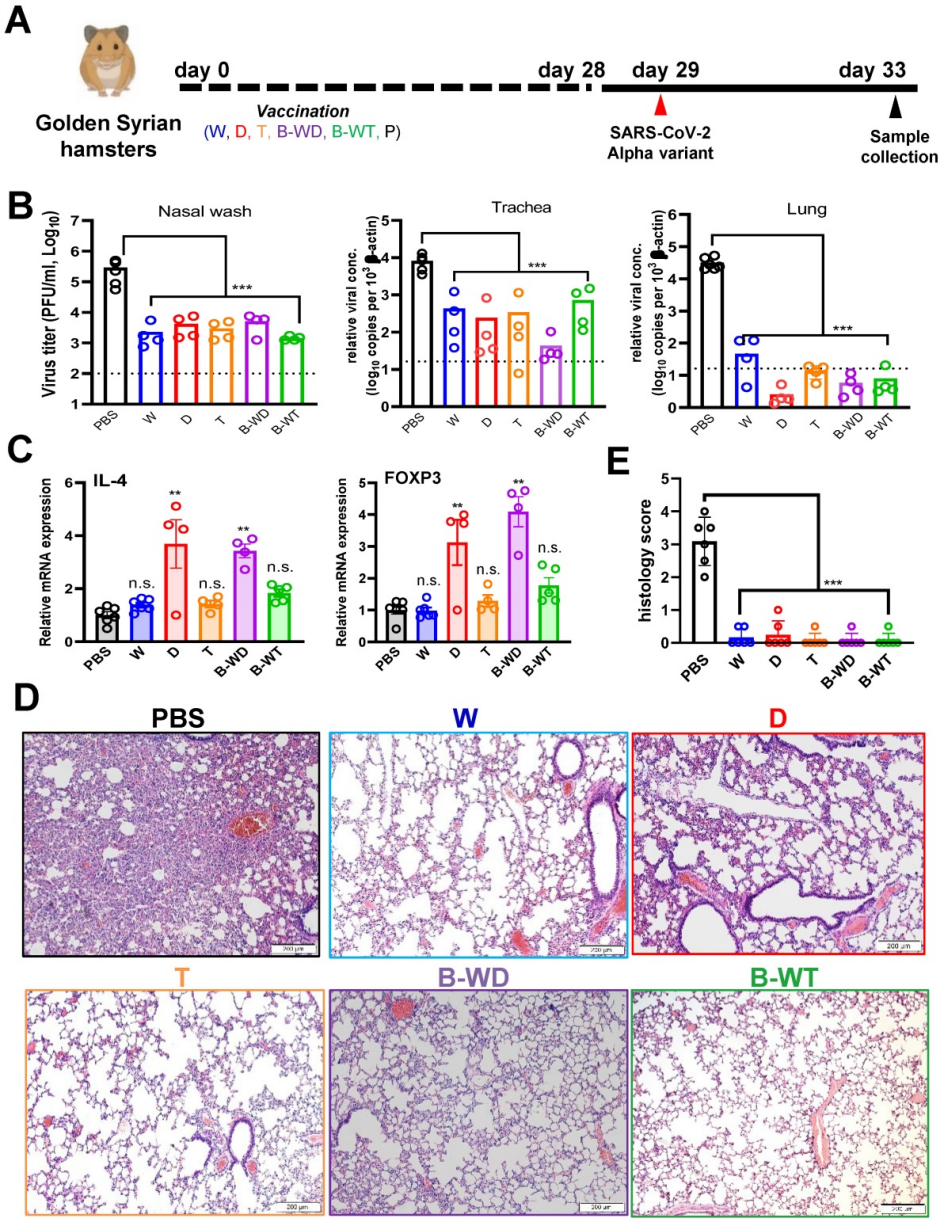
Vaccination with the indicated inactivated SARS-CoV-2 strains confers protection to infection of hamster lungs with SARS-CoV-2 Alpha variant. (**A**) Virus challenge scheme. Vaccinated hamsters were intranasally inoculated with 10^5^ PFU (in 50μl) of SARS-CoV-2 Alpha variant. At 4 dpi (i.e. 33 days after vaccination), viral titers in hamster nasal wash were quantitated by RT-qPCR, whereas tracheal and lung samples were analysed by RT-qPCR (**B**). Broken lines in bar plots indicate detection limit. (**C**) Proinflammatory cytokine and chemokine gene expression in Alpha-infected hamsters. Transcripts of representative chemokines and cytokines in the lung tissue homogenates of the indicated groups were quantitated by RT-qPCR. Results are shown as means ± SEM. Statistical analysis was performed by Student's t test (n.s.: not significant; *: P < 0.05; **: P < 0.01; ***: P < 0.001). (**D**) Impact of vaccination on histopathological changes in lungs of SARS-CoV-2 Alpha variant-infected hamsters. Lung histopathological changes of each vaccinated hamster group at 4 dpi. Representative lung tissue sections were stained with hematoxylin and eosin. Bar, 200 μm. (**E**) Pathological changes in Alpha-infected lungs from the indicated vaccinated groups (**: P < 0.01).

To determine whether our findings on the Delta variant are applicable to other VOCs, we extended our analysis of the protective efficacies of vaccines in hamsters to the Alpha variant (Figure 5A), which was a globally prevalent VOC in the early phase of the pandemic before the emergence of the Delta strain 5, 9. Results from plaque assay and RT-qPCR indicated that viral loads of the nasal wash, tracheal and lung tissues of the vaccinated hamsters decreased substantially (Figure 5B). Expression levels of IL-4 mRNA significantly increased in the D and B-WD groups, whereas FOXP3 mRNA expression was elevated in the D and B-WD groups (Figure 5C). Histology results showed notable amelioration in lung damage in all groups (Figure 5D, E). Thus, all tested inactivated vaccines remained efficacious in preventing infection and replication of the Alpha variant as well as the associated tissue damage.

## Discussion

All inactivated vaccines tested in this study were capable of successfully inducing RBD-specific humoral responses in hamsters which plateaued at day 21 (Figure 1). Satisfactory neutralization results were observed upon challenge with the ancestral Wuhan strain of SARS-CoV-2 and other VOCs, indicating cross-variant protection (Figures 1). Among all three monovalent vaccines, weak cross-variant protection was seen when challenged with the Delta variant (Figure 4). This variant is modestly vaccine-evasive 33, which is likely due to mutations in the RBD, a critical determinant of protection and immune evasion 33, 40. Compared to the ancestral Wuhan strain, nine point mutations are found in the spike region of this variant, including two in the RBD (Figure S1). Particularly, the L452R substitution is crucial in conferring resistance to neutralizing antibodies 41-43. Our results suggested that even for the Delta variant, a variant-specific inactivated vaccine might be required for optimal protection (Figure 4). In terms of immune protection, the W-vaccinated group was deemed the weakest, as evidenced by residual pathological features and inflammatory response in lung sections (Figure 3). This lends further support to the notion that a next-generation variant-targeting inactivated vaccine is necessary. Notably, the neutralizing activity of all tested inactivated vaccines was weak in the nasal cavity (Figure 4B), suggesting that they might be suboptimal in blocking SARS-CoV-2 transmission. This is consistent with the general features and limitations of inactivated vaccines as well as their performance in the real world 2, 19, 44. The monovalent D and the bivalent B-WD vaccines seem to offer relatively stronger homotypic protection against the Delta variant in the upper respiratory tract (Figure 4B), highlighting the benefits of variant-specific inactivated vaccines. Before variant-specific vaccines are available, booster injection with a mRNA, adenoviral vectored or protein subunit vaccine in previous recipients of two doses of inactivated vaccines is highly immunogenic and provides better protection against VOCs 16, 40, 44-46. However, for optimal protection in the upper respiratory tract, mucosal immunity is required 19. In this regard, nasal administration of live attenuated vaccines and influenza virus-based vaccines expressing SARS-CoV-2 spike protein as either primary or booster immunization might provide the desired effect 47-49. It will be of interest to see whether these vaccines might be good inducers of mucosal immunity and secretory IgA in the upper respiratory tract.

As seen in the results of microneutralization assays (Figure 1E-H and Figure S4), limited protection was observed for all vaccinated groups challenged with the Omicron variant. This is consistent with the notion that the Omicron variant is one of most immune-evasive among all existing VOCs 13, 15-18. Interestingly, monovalent T and bivalent B-WT inactivated vaccines based on the Theta variant exhibited relatively good protection against challenge with the Omicron variant (Figure 2). Both the Theta and Omicron variants not only harbor the N501Y mutation but are also mutated at E484 (Figure S1). These point mutations within the RBD are known to be critical to neutralization 6, 9, 42, 50 and they likely account for the cross-variant protection between the Theta and Omicron variants. The vaccine evasiveness of the Omicron variant necessitates the development of an Omicron-specific inactivated vaccine. We are in the process of assessing whether such a vaccine offers the best protection against the Omicron variant. Due to the low viral titer of the Omicron variant, we have encountered some difficulties in mass producing the Omicron-specific inactivated vaccine and more time is required for problem solving. Before various types of Omicron-specific vaccines are available for human use, booster injection with existing non-Omicron-specific vaccines remains the sub-optimal and pragmatic way of gaining some protection against the Omicron variant 44, 45.

There is a tug-of-war between the host immunity and SARS-CoV-2 51. It would be ideal to develop a universal vaccine that is capable of protecting against all emerging and/or circulating SARS-CoV-2 strains. In addition to targeting the conserved regions of SARS-CoV-2 52, inclusion of all critical strains in a multivalent vaccine is another strategy for the development of a universal vaccine. The two bivalent vaccines tested in our study performed better in eliciting protective humoral responses and alleviating damage in respiratory tissues upon infection with Alpha and Delta variants (Figures 4 and 5). Bivalent and multivalent vaccines are not uncommon. One of the most iconic examples is the human papillomavirus (HPV) vaccine, which contains nine high-risk HPV strains in a single vaccine to maximize the coverage of HPV for prophylactic measures against HPV infection and development of cervical cancer 53. Inactivated vaccines for influenza viruses are also trivalent or quadrivalent 54. By the same token, an inactivated SARS-CoV-2 vaccine containing more than one prevalent strain should be considered. Addition of new and emerging strains to bivalent or multivalent vaccines would be necessary. In addition to the cross-variant protective effect of the inactivated Theta virus against the Omicron variant discussed above, immunization with D and B-WD vaccines was shown to protect against challenge with the Alpha variant (Figure 5). Thus, careful selection of some representative variants might be sufficient for the development of a universal inactivated vaccine against SARS-CoV-2. The Omicron variant is currently predominant in many parts of the world and its inclusion in the inactivated vaccine is therefore desired. Addition of another more distant and once prevalent variant such as the Delta variant might further improve the breadth of protection.

A significant portion of the world's population has already been vaccinated against the ancestral version of SARS-CoV-2. Natural infection including breakthrough infection has also contributed substantially to the strength and breadth of herd immunity. In this context, booster vaccination with one or more prevalent strains carrying new adaptive mutations should be an ideal approach. Although this study was conducted with inactivated vaccines, in principle, the concepts of developing multivalent vaccines against SARS-CoV-2 and targeting circulating VOCs in booster vaccination should also apply in other types of vaccines including mRNA vaccines, viral vectored vaccines and protein subunit vaccines.

## Materials and Methods

### Generation of inactivated virus

African green monkey kidney Vero-E6-TMPRSS2 cells were cultured in Dulbecco's modified Eagle's medium (DMEM) containing 10% fetal bovine serum, 1 µg/mL G418, and 1% penicillin/streptomycin at 37^o^C until 90% confluency. Vero-E6-TMPRSS2 cells were used since this cell line allows better viral propagation 55. Moreover, SARS-CoV-2 might be genetically more stable and fewer sequence variations were observed in virus populations in Vero-E6-TMPRSS2 cells than in Vero cells 56. Cells were then trypsinized and transferred to 4-layer cell culture discs. The cells were infected with desired strains of SARS-CoV-2 on the following day and incubated at 37^o^C for 5 days. Virus-containing supernatant was harvested and inactivated using 0.2% paraformaldehyde solution in PBS for another 5 days. Cytopathic effect (CPE) was monitored to validate complete inactivation using Vero-E6-TMPRSS2 cells. In the absence of any observable CPE, the supernatant was syringe-filtered at 0.45 μm, cushioned with 30% sucrose solution in PBS and centrifuged at 80,000×g for 2 h at 4^o^C. The resulting pellet was resuspended in PBS, cushioned with 30%, 50% then 70% sucrose solution, and subjected to centrifugation at 120,000 ×g at 4^o^C overnight. Approximately 1 mL fraction was collected between the 30/50 sucrose interface, diluted in PBS and centrifuged at 120,000 ×g at 4^o^C for 2 h. The pellet was then resuspended with PBS, stored at -80^o^C until further use 57. Viruses for generating inactivated vaccines have recently been reported 13, 42. Each inactivated vaccine (3 µg/dose) was injected to hamster followed by one booster of the same dose of vaccine (3 µg) 2 weeks after primary injection. The bivalent vaccines were formulated by mixing equal amount of the two inactivated vaccines (i.e., 1.5 µg of vaccine W + 1.5 µg of vaccine D or T).

### ELISA

Flat-bottom 96-well plates (Costar) were coated with 100 μL of SARS-CoV-2 spike RBD protein (0.5 μg/mL) at 4^o^C overnight. After three washes with 0.05% Tween-20 in PBS, the coated antigen was blocked with 3% bovine serum albumin (BSA, Sigma-Aldrich) in PBS for 1 h, and incubated with 100 μL of serially diluted sera in 0.1% BSA in PBS for 1 h at 37^o^C. After washing, plates were incubated with anti-hamster IgG-horseradish peroxidase (HRP) conjugate (diluted 1:2000 in 20% goat-serum in 0.05% PBS-T) for 1 h at 37^o^C. After four further washes, 3,3',5,5'-tetramethylbenzidine (TMB, Thermo Scientific) was added to each well for detection. Followed by 15-min incubation at room temperature (RT), 1M HCl was added as a stopping reagent. Optical density at 450 nm (OD450) was measured using a microplate reader 58.

### Microneutralization assay

Live virus microneutralization assay was performed as we described previously 58. Briefly, serum specimens were serially diluted in 3-folds from 1:10. Equal volumes of serially diluted sera and the desired SARS-CoV-2 strains (1 × 10^3^ PFU/mL) were mixed, added onto 96-well plates and incubated for 1 h at 37^o^C. Mixtures were then transferred to 96-well plates pre-seeded with 2 × 10^4^/well Vero E6 cells and incubated at 37^o^C overnight. The culture medium of the plates was removed, air-dried in a biosafety cabinet (BSC) for 20 min. Cells were then fixed with 4% paraformaldehyde solution. To further permeabilize the cells, 100 μL of 0.2% Triton X-100 in PBS was added to each well and the plates were incubated at RT for 15 min. After three washes in PBS, the plates were incubated with cross-reactive rabbit anti-SARS-CoV-2 N IgG (diluted 1:4000 in 0.2% Triton X-100 in PBS) for 1 h, followed by the addition of Alexa Fluor 488 goat anti-rabbit IgG (H+L) cross-adsorbed secondary antibody (diluted 1:1000 in 0.2% Triton X-100 in PBS, Life Technologies) and incubation at RT for 1 h. The plates were analyzed using Sapphire Biomolecular Imager (Azure Biosystems). The numbers of SARS-CoV-2 foci were quantified using custom-built ReadPlate 3.0 plugin in ImageJ 59.

### Surrogate virus neutralization test (sVNT)

sVNT was performed using cPass (Genscript) according to manufacturer's instructions with slight modifications 33. Briefly, hamster sera were diluted 1:10 and equal volumes of diluted sera were incubated with HRP-conjugated RBD for 30 min. The mixture is added to ACE2-coated ELISA plate for 30 min at 37°C. Colorimetric signals were developed using TMB substrate and the reaction was stopped with 1 M HCl. Absorbance at 450 nm was measured using a microplate reader.

### Infection of golden Syrian hamsters

All protocols for animal experiments have been approved by the Committee on the Use of Live Animals in Teaching and Research (CULATR) of the University of Hong Kong (HKU). They were also performed in accordance with the biosafety level 3 animal facilities standard operating procedures (Reference code: CULATR 5754-21). SARS-CoV-2 virus HKU-001a isolate (GenBank accession number: MT230904) representing the ancestral Wuhan strain and designated WT in this study was isolated from the nasopharyngeal aspirate specimen of a laboratory-confirmed COVID-19 patient in Hong Kong. It was cultured and titrated in Vero-E6-TMPRSS2 cells by plaque assays. The other SARS-CoV-2 strains originated from Philippines, England and India and used in this study have been described previously 13, 42.

Male and female golden Syrian hamsters, aged 4-6 weeks old, were obtained from the Chinese University of Hong Kong Laboratory Animal Service through the Center for Comparative Medicine Research of the HKU. They were kept in Biosafety Level-2 facility with access to standard pellet feed and water *ad libitum*. Baseline body weights were recorded before infection. PBS was used to dilute virus stocks to the desired concentration. Prior to vaccination, the inactivated viruses or PBS were mixed with the aluminum adjuvant (Thermo Scientific Cat#77161) at a ratio of 1:1. At day 29 post-vaccination, the anesthetized hamsters were intranasally challenged with 10^5^ PFU (in 50μl) of SARS-CoV-2. For virological and histopathological examinations, 4 hamsters per group were sacrificed at 4 dpi and their nasal wash, tracheae, lung and blood samples were collected for analyses. One half of the lung tissue was used for virus titration by RT-qPCR method, while the other half was immediately fixed in 10% of PBS-buffered formaldehyde for histopathological analyses. Tissue slides were examined in a single-blind manner 58. The level of inflammation was scored as follows: 0, no inflammation; 1, perivascular cuff of inflammatory cells; 2, mild inflammation; 3, moderate inflammation; 4, severe inflammation involving over one half of the lung.

### Real-time quantitative PCR (qPCR) analysis of cytokines in lung

A total of 1 μg lung tissue RNA was reversely transcribed using TAKARA PrimeScript^TM^ reverse transcription reagent kit with gDNA eraser according to manufacturer's instructions. cDNA was diluted 1:10 and stored at -20^o^C until further use. All real-time quantitative PCR (qPCR) reactions were carried out in duplicate using the CFX96 C1000 real-time thermal cycler (BioRad). A 25 μL reaction contained 2 μL of diluted cDNA template, 1× TB Green Premix Ex Taq and gene-specific forward and reverse primers. The levels of gene expression were determined by normalizing to housekeeping genes; the relative levels of the transcripts were determined using the 2^-ΔΔCT^ mathematical model 60.

## Supplementary Material

Supplementary figures.
