# Supplementary material for: Cross-variant protection against SARS-CoV-2 infection in hamsters immunized with monovalent and bivalent inactivated vaccines
Source: Int J Biol Sci. 2022 Jul 13;18(12):4781–91. doi: 10.7150/ijbs.72109 (PMC9305277; doi:10.7150/ijbs.72109)
Supplement: Supplementary file 1 — Supplementary figures. [file ijbsv18p4781s1.pdf]

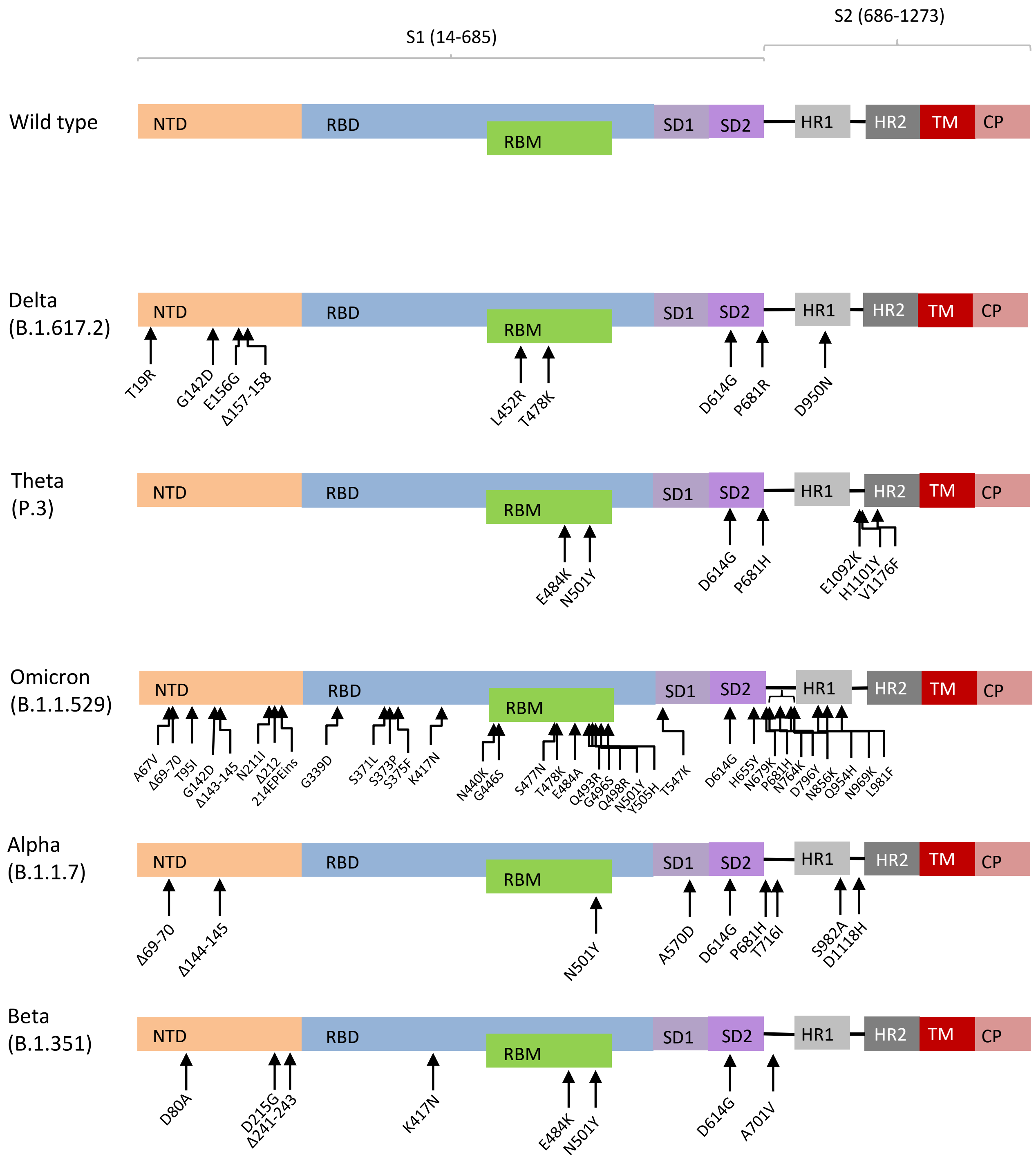

Figure S1. Mutations in S protein of SARS-CoV-2 VOCs. Schematic depiction of the locations of amino acid substitutions in the indicated four VOCs. Receptor binding domain (RBD) is shown in blue. N-terminal domain (NTD) is in orange. RBM: receptor-binding motif. SD1: subdomain 1. SD2: subdomain 2. HR1: heptad repeat 1. HR2: heptad repeat 2. TM: transmembrane domain. CP: cytoplasmic domain.

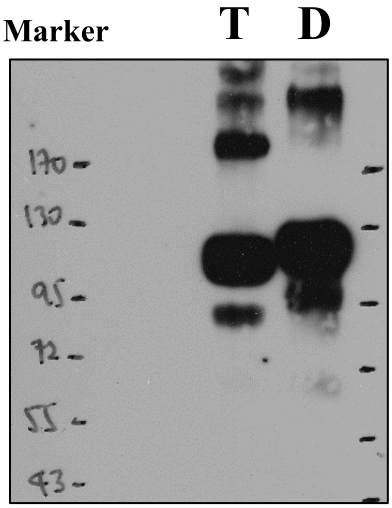

Figure S2. Expression of spike protein from the indicated SARS-CoV-2 strains by Western blotting. Lysates of Vero-E6-TMPRSS2 cells infected by the indicated strains were probed by anti-SARS-CoV-2 S antibodies.

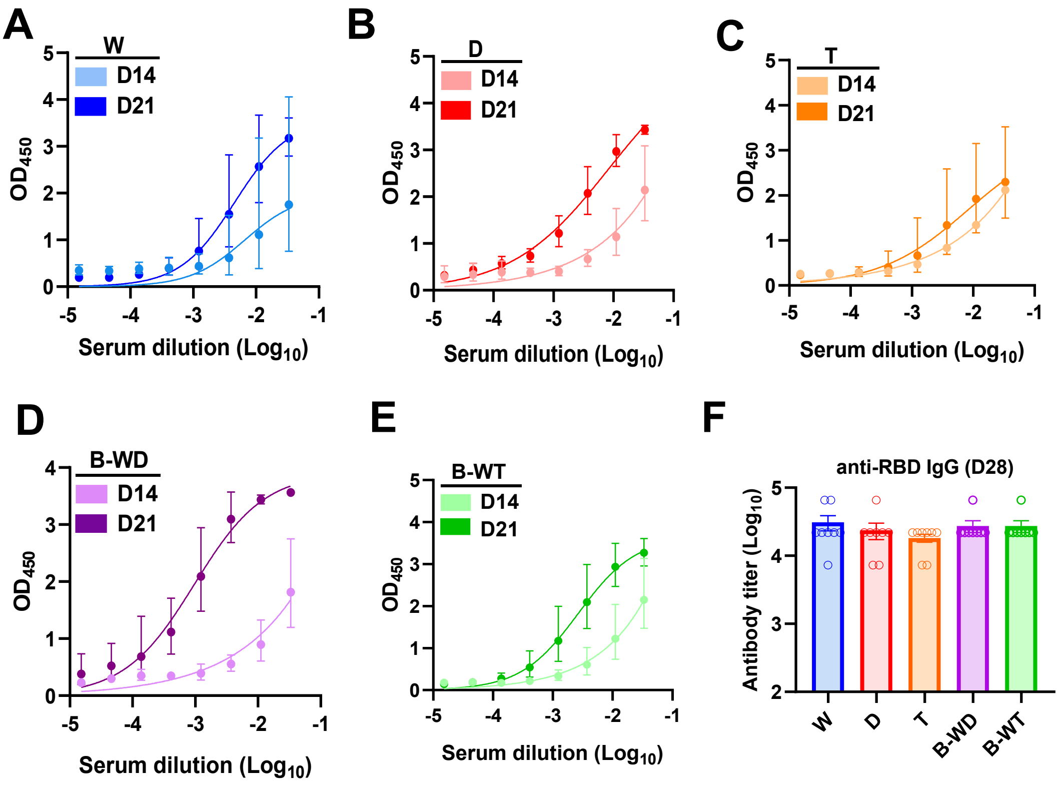

Figure S3. ELISA analysis of antibodies against SARS-CoV-2 RBD for the indicated groups collected at days 14 (D14), 21 (D21) and 28 (D28) post-vaccination. (**A-E**) IgG antibodies against SARS-CoV-2 RBD in hamsters immunized with the indicated vaccines detected by ELISA. (**F**) Endpoint titer of antibodies against SARS-CoV-2 RBD for the indicated groups collected at day 28 (D28) post-vaccination as detected by ELISA.

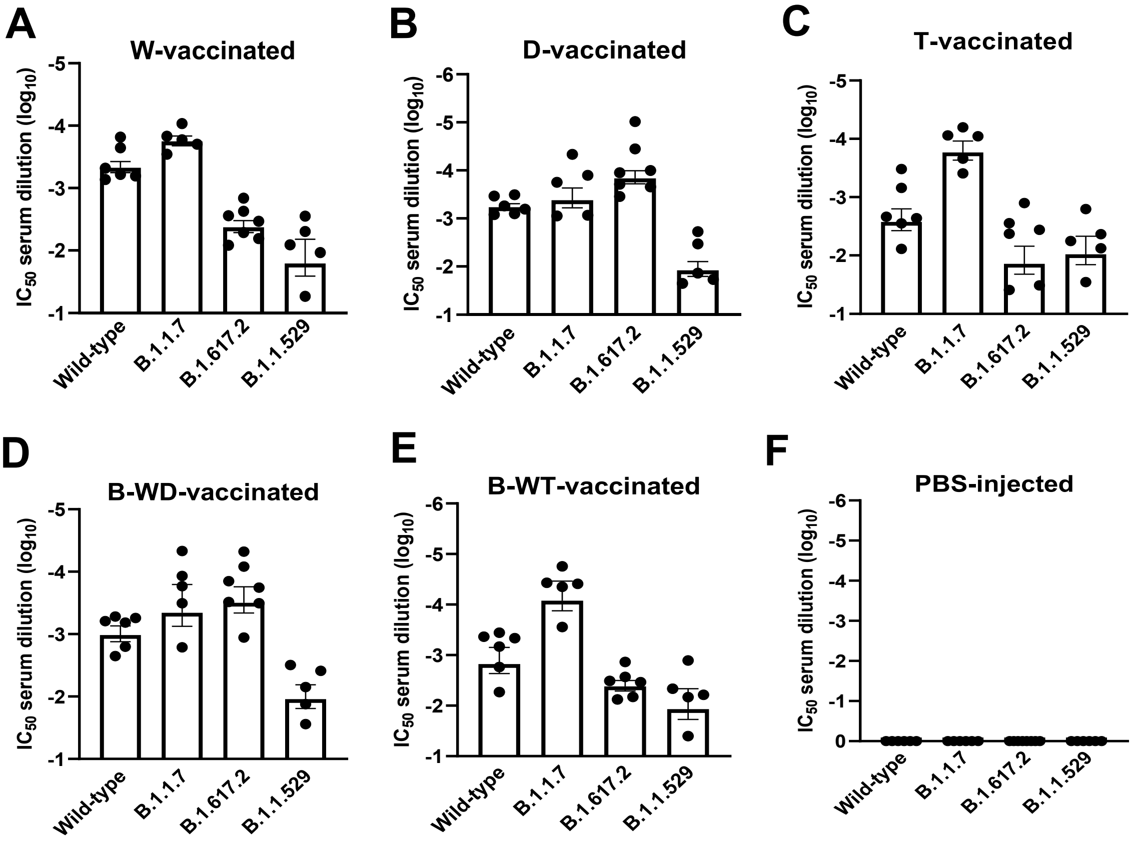

Figure S4. Neutralization activity of the serum samples collected at day 28 post-vaccination from hamsters immunized with the indicated types of inactivated vaccines detected by microneutralization assay.
